# The Effects of Digital eHealth Versus Onsite 2-Day Group-Based Education in 255 Patients With Irritable Bowel Syndrome: Cohort Study

**DOI:** 10.2196/43618

**Published:** 2025-02-03

**Authors:** Birgitte Berentsen, Camilla Thuen, Eline Margrete Randulff Hillestad, Elisabeth Kjelsvik Steinsvik, Trygve Hausken, Jan Gunnar Hatlebakk

**Affiliations:** 1 Department of Clinical Medicine, University of Bergen, Bergen, Norway; 2 Department of Medicine, National Center for Functional Gastrointestinal Disorders, Haukeland University Hospital, Bergen, Norway

**Keywords:** irritable bowel syndrome, IBS, eHealth, internet-guided, patient education, self-management, self-reported, patient behavior, quality of life, QOL, anxiety, depression, gastrointestinal, physiotherapist, kinesiology, cognitive behavioural therapy, CBT, Hospital Anxiety and Depression Scale, HADS, client satisfaction questionnaire, CSQ, Mann-Whitney U test, nonparametric, Wilcoxon test, neurogastroenterology

## Abstract

**Background:**

Irritable bowel syndrome (IBS) has a high worldwide prevalence and there are few effective treatment options. Patient education can influence patient behavior that subsequently may lead to changes in attitudes and skills necessary for maintenance or improvement in management of symptom severity and quality of life. However, as postdiagnostic patient education can be resource demanding, assessment of digital approaches and verification of their effectiveness is warranted.

**Objective:**

This cohort study aimed to investigate the effects of a digital web-based multidisciplinary eHealth program on the domains of symptom severity (Irritable Bowel Syndrome Symptom Severity Scale [IBS-SSS]), quality of life (irritable bowel syndrome quality of life [IBS-QOL]), anxiety and depression (Hospital Anxiety and Depression Scale), and a measure of general client satisfaction (client satisfaction questionnaire), compared with an onsite multidisciplinary 2-day group-based education program (“IBS-school”), in 2 cohorts of 255 patients with IBS.

**Methods:**

Patients diagnosed with IBS, aged 15-70 years, were enrolled after referral to the Section of Gastroenterology at Haukeland University Hospital, Norway. In total, 132 patients were recruited to the eHealth program and 123 to the IBS-school group for comparison. Data were self-reported and collected digitally at enrollment and after 3 months, between 2017 and 2019. Furthermore, 71 attending the eHealth program and 49 attending the IBS-school completed the questionnaires at 3 months. Intervention response was defined as a reduction of ≥50 points on the IBS-SSS.

**Results:**

Patients attending the eHealth program reported a significant reduction in IBS symptom severity 3 months after treatment (n=71), compared with patients attending the IBS-school (n=50). Overall, patients categorized as intervention responders in both programs showed a significant reduction in symptom severity at 3 months. Here, 41% (29/71) of patients attending the eHealth program reported a mean IBS-SSS reduction of 103 (SD 72.0) points (*P*<.001). In addition, these patients reported reduced anxiety (*P*>.001) and depression (*P*=.002) and enhanced quality of life (*P*=.03), especially the degrees of dysphoria, body image, food avoidance, health worry, interference with activity, relations, and social relations. Patients responding to the IBS-school intervention (18/50, 36%) reported a mean IBS-SSS reduction of 119 (SD 86.2) points (*P*<.001), and reduced depression scores (*P*=.046), but no difference in overall quality of life. Both groups reported the respective interventions as “good” quality health care programs, scoring them 23.5 (SD 4)—the eHealth program 23.5 (SD 4), and the IBS-school 24.2 (SD 4)—on the client satisfaction questionnaire.

**Conclusions:**

We conclude that the digital multidisciplinary eHealth program has a significant effect on IBS symptom severity in a portion of patients; it is useful as a tool in disease self-management and does not result in worse symptom scores than an onsite multidisciplinary 2-day group-based education program after 3 months. We believe these results indicate that a digital eHealth approach is preferable to an onsite multidisciplinary 2-day group-based education program covering the same topics.

## Introduction

Irritable bowel syndrome (IBS) is a chronic disorder manifested by recurrent abdominal pain and alterations in stool form or frequency [1,2]. The condition affects between 4% and 9.2% of the global population [3-5] and it is highly heterogenous. IBS’ unclear etiology involves multifactorial disturbances of the bidirectional communication between the gut and the brain, including visceral hypersensitivity, low-grade inflammatory responses, intestinal motility disturbances, alterations of central nervous system processing, and alterations in gut microbiota composition [6]. However, no clear biomarker or therapeutic target for IBS has been identified. The condition lacks both a cure and medication that gives sufficient symptom relief, a fact that highlights the necessity of integrating nonpharmacological approaches including patient education in patient care [7]. Patients with IBS require personalized treatment for successful symptom relief. Approaches may include physical therapy, cognitive behavioral therapy (CBT), hypnotherapy, mindfulness and exposure therapy, and comprehensive dietary guidance by registered dietitians such as the low FODMAP (fermentable oligosaccharides, disaccharides, monosaccharides, and polyols) diet. However, access to these treatment options is often limited due to lack of trained professionals, travel distances, and cost. Thus, more accessible treatment options are warranted. Web-based interventions, patient education, and self-management has been demonstrated to be effective in patients suffering from chronic diseases, including IBS [8-10]. A systematic review from 2017 concluded with mixed results regarding the effectiveness of web-based mindfulness-based interventions compared with active control treatment conditions such as CBT. However, the study showed that treatment targeting symptoms of IBS had the largest effect size improvements [11]. A longitudinal qualitative study from 2020 showed that both telephone-based CBT and web-based CBT for IBS were positively received and had lasting positive impacts on participants’ understanding of IBS symptoms, quality of life, and IBS-related behaviors [12]. A recent Japanese randomized controlled trial has shown that a multidisciplinary eHealth self-management program can reduce the severity of IBS-symptoms and improved the quality of life [13].

Indeed, limited health care resources, national priority guidelines, and sparse treatment options may restrict any long-term follow-up that patients with IBS may request from a secondary or tertiary care institution. Since 2012, Haukeland University Hospital has offered patients with IBS an onsite multidisciplinary 2-day group-based education program, a so-called “IBS-school.” The multidisciplinary approach is designed to provide patients with evidence-based information and practical skills that may lead to improved IBS management, reduced IBS symptoms, and enhanced quality of life. Based on our clinical experience with group education, we have developed a novel internet-guided multidisciplinary self-care management program for patients with IBS, from now on referred to as the “eHealth program.” The content in the eHealth program is based on topics covered in the IBS-school, but the patients also have access to digital clinical support during the program. Herein, we hypothesized that the eHealth program would have an equally good effect on symptom severity, quality of life, and patient satisfaction, compared with a cohort of patients attending IBS-school. Data from neither program have been published before. In this study, we aimed to investigate the effects of a digital web-based multidisciplinary eHealth program on the domains of symptom severity (Irritable Bowel Syndrome Symptom Severity Scale [IBS-SSS]), quality of life (irritable bowel syndrome quality of life [IBS-QOL]), anxiety and depression (Hospital Anxiety and Depression Scale [HADS]), and a measure of general client satisfaction (client satisfaction questionnaire [CSQ-8]), compared with an onsite multidisciplinary 2-day group-based education program, IBS-school, in 2 cohorts of 255 patients with IBS.

## Methods

### Patient Sample, Randomization, and Treatments

**Figure 1.**
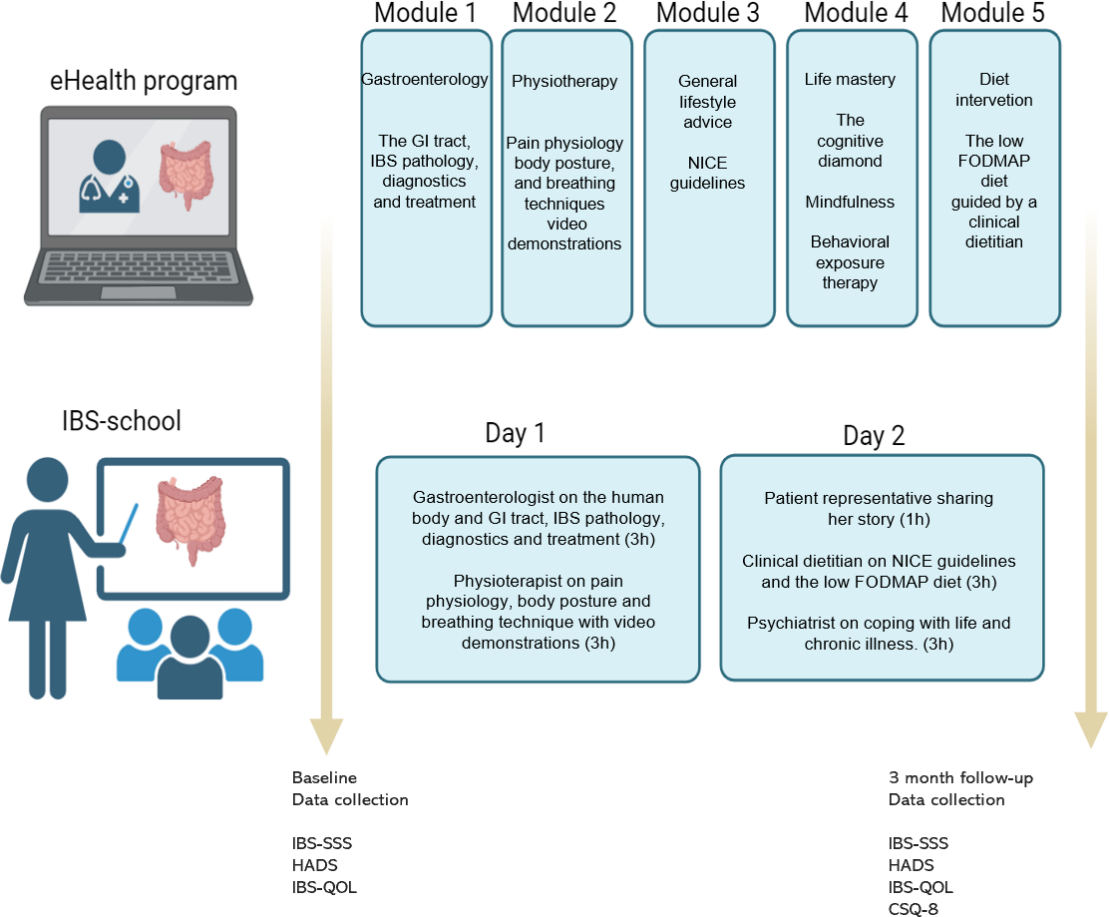
Intervention overview. CSQ-8: client satisfaction questionnaire; FODMAP: fermentable oligosaccharides, disaccharides, monosaccharides, and polyols; GI: gastrointestinal; HADS: Hospital Anxiety and Depression Scale; IBS: irritable bowel syndrome; IBS-QOL: irritable bowel syndrome quality of life; IBS-SSS: Irritable Bowel Syndrome Symptom Severity Scale; NICE: National Institute for Health and Care Excellence.

In this study**,** 255 patients between 15 and 70 years were recruited after being accepted for patient education at Haukeland University Hospital in Norway between 2017 and 2019. Random patients on the waiting list to the onsite multidisciplinary 2-day group-based education program, IBS-school, were contacted by phone by a study nurse and offered to attend the novel multidisciplinary digital 5-module eHealth program, from here on referred to as the “eHealth program.” Patients attending the IBS-school were recruited on site when attending the 2-day group-based education program. Patients received oral and written information before giving written consent and sent it to the hospital by post. Furthermore, 123 patients that attended the IBS-school were used for comparison (N=255, 1:1). Here, patients were recruited by a study nurse on site. They received an oral and written information about the study before signing consent (Figure 1). Inclusion criteria included (1) being referred to receiving patient education on IBS by a gastroenterologist or general practitioner after the diagnosis of IBS had been determined (*International Classification of Primary Care, Second Editon* [*ICPC-2*] code D93; *International Statistical Classification of Diseases, Tenth Revision* [*ICD-10*] code K58), (2) being between 18 and 70 years, and (3) understanding written and oral Norwegian. There were no specific exclusion criteria.

### Ethical Considerations

Eligible patients gave informed written consent and participants were given the option to withdraw from the study at any time point without a specific reason. The data were deidentified and stored on a secure hospital server, and analysis was performed on anonymous data. None of the participants were compensated. The study was approved by the Regional Ethical Committee of Western Norway (REC-2016/1098).

### Interventions

#### The Irritable Bowel Syndrome–School

Patients attended an onsite multidisciplinary 2-day group-based education program, IBS-school, at Haukeland University Hospital. The number of participants varied between 15 and 40 each month. The program involved lectures and question and answer sessions by 4 health care professionals. Refer to Figure 1 for the intervention outline. On day 1, a gastroenterologist talked about the human body and the gastrointestinal system, what IBS is, what causes it, diagnostics, and treatment options (3 hours). Second, a physiotherapist was giving a lecture on body posture and breathing techniques including demonstrations, introducing pain physiology, and the function of the human nervous system (3 hours). On day 2, a patient representative shared her personal experience with IBS (1 hour), and a clinical dietitian gave a lecture on National Institute for Health and Care Excellence (NICE) guidelines [14] that summarizes the most recent recommendations on IBS in adults in primary care, and the low FODMAP diet [15] (3 hours). Finally, a psychiatrist gave a lecture on coping with life including health worry, social relations, tiring thoughts and feelings, adaptations, and symptoms. A nurse with specialization in gastroenterology hosted the group education program to create a comfortable environment for exchange of personal experiences and participate in group assignments.

#### The eHealth Program

Patients who were enrolled in the digital eHealth program had access to a comprehensive, multidisciplinary web-based program that consisted of 5 modules (refer to Figure 1 for the outline). The modules consisted of instructive texts, videos, animations, and images over 150 web-pages on a digital treatment platform by CheckWare AS [16]. In addition, patients carried out “home assignments” based on principles of CBT, a protocol by Ljótsson et al [17] at the Swedish Karolinska Institute, followed by an optional low FODMAP diet intervention guided by a clinical dietitian. The eHealth program allowed a secure login (eg, bank-ID) and digital communication between patient and a clinical dietitian throughout the process, at the patient’s own request and need. The patients were expected to finish the program over a period of 3‐12 weeks, at his or her own pace, all dependent on their motivation and work capacity. Module contents have been described further in this study. Module 1 describes the body and the gastrointestinal system. In this first module, the patient gets an introduction to what IBS is, how it is diagnosed, and what causes it. The patient learns about the function of our digestive system and how it is regulated, and how it can be disturbed in people with IBS. Module 2 describes the posture and breathing techniques. This module focuses on the connection between IBS and musculoskeletal disorders. Many people with IBS may have a “hyperactive” nervous system that can cause pain and physical maladjustments. In this module, a physiotherapist introduces the patient to pain physiology and how the nervous system works. There is a practical section with useful exercises for people with IBS. The module will give the patient an understanding of how long-term pain occurs, why it often persists, and how to influence it. Module 3 consists of diet and lifestyle advices. There is no miracle cure for IBS, but many people experience improvement by following some general advice. In this module, we look at lifestyle advice that has been shown by research to improve symptoms in people with IBS. It is recommended to try this before eliminating other foods or following strict diets. Module 4 focuses on coping with life. In this module, the patient learns techniques from cognitive therapy including “the cognitive diamond,” mindfulness, and practice systematic exposure exercises has previously shown beneficial effects for patients with IBS. The protocol has been described in a study by Ljótsson et al [17]. Module 5 describes the dietary intervention with a low FODMAP diet. This is a dietary treatment that provides symptom relief in approximately 70% of patients with IBS [18]. In this module, the patient will be introduced to the low FODMAP diet in both theory and practice. The patient had access to digital guidance by a clinical dietitian during the entire course of the study.

### Questionnaires

#### Overview

Patients completed questionnaires related to IBS symptom severity (IBS-SSS) [2], quality of life, (IBS-QOL) [19], and anxiety and depression (HADS) [20] upon enrollment at baseline and after 3 months.

The IBS-SSS is considered the gold standard measure of IBS symptoms and contains 5 questions that measure the frequency of abdominal pain, the severity of abdominal distention, dissatisfaction with bowel habits, and interference with quality of life, scored in the range of 0‐500. A higher score indicating worse condition, scores <175 represent mild IBS symptoms, 175‐300 represents moderate severity, scores >300 represent severe IBS [2].

The IBS-QOL is a condition-specific measure for assessing health-related quality of life in IBS. It consists of 34 items, each with a 5-point response scale in a range of 0‐100, where the higher score indicates a better IBS specific quality of life. There are 8 subscale scores for the IBS-QOL: dysphoria, interference with activity, body image, health worry, food avoidance, social reaction, sexual, and relationships [21].

HADS is a scale of 14 items designed to measure anxiety and depression, 7 items for each subscale (ie, anxiety and depression). The total score is the sum of the 14 items, and for each subscale the score is the sum of the respective 7 items that range from 0 to 21 [20].

CSQ-8 [22] was completed at 3 months. The questionnaire is an 8-item measure with a total score ranging from 8 to 32, with the higher number indicating greater satisfaction with treatment.

#### Primary Outcome Measure and Definition of Intervention Responders

In the field of IBS research, a change of 50 points in IBS-SSS score has shown to reliably indicate improvement in IBS symptom severity [2]. Our primary outcome measure was a >50-point reduction in IBS-SSS at 3 months, compared with baseline. Patients reporting a ≥50-point reduction in IBS-SSS at 3 months were categorized as “responders” to the intervention. Patients reporting <50-points on IBS-SSS were categorized as “nonresponders” to the intervention.

### Statistical Analysis

Statistical analyses were performed using SPSS Statistics (version 26, IBM) for Microsoft Windows. Baseline patient characteristics and questionnaires were first presented according to intervention—eHealth group and IBS-school. At 3 months after intervention, patients were categorized as responders or nonresponders. For comparison between groups, unpaired *t* test were performed for parametric data and Mann-Whitney *U* for nonparametric data. For comparisons before and after interventions, paired *t* tests were performed for parametric data and Wilcoxon signed rank test for nonparametric data. A *P* value <.05 was considered statistically significant. The intervention responder or nonresponder analysis to the eHealth program or IBS-school was carried out performing a paired *t* test on data from patients who filled out the 3-month questionnaires (n=71 and n=50, respectively). A *χ*^*2*^ test of independence was used to assess differences between intervention response.

## Results

### Patients and Characteristics

**Figure 2.**
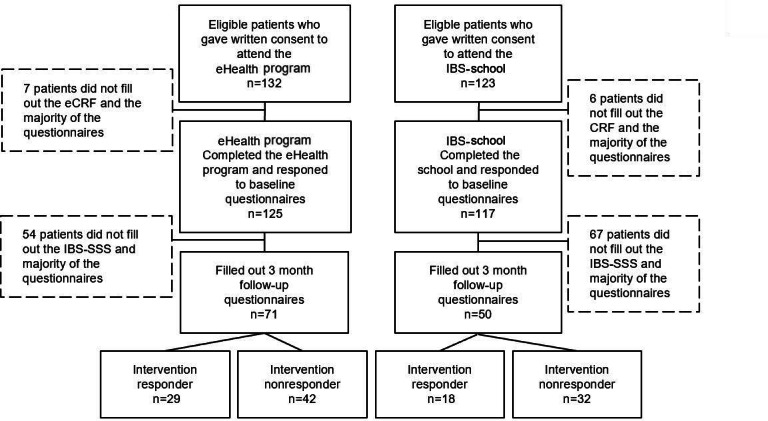
Flow chart of participating patients with IBS. Patients reporting a ≥50-point reduction in the IBS-SSS at 3 months were categorized as “responders to the intervention”. Patients reporting <50-points on the IBS-SSS were categorized as “nonresponders to the intervention”. CRF: case report form; eCRF: electronic case report form; IBS: irritable bowel syndrome; IBS-SSS: Irritable Bowel Syndrome Symptom Severity Scale.

A flowchart of participating patients is illustrated in Figure 2. Of the 132 eligible patients who gave written consent to participate in the eHealth program group of the study, 7 did not fill out the minimum requirement including the electronic case report form and the IBS-SSS questionnaire. Of the 125 patients who completed the program, 54 patients did not respond to the IBS-SSS questionnaire at 3 months. Hence, the follow-up data from patients attending the eHealth program reduced to n=71 at 3 months. Of the 123 eligible patients who gave written consent to participate in the IBS-school group of the study, 5 did not fill out the minimum requirement including the electronic case report form and the IBS-SSS questionnaire. Thus, of the 118 patients who completed the program, 69 patients did not respond to the IBS-SSS questionnaire at 3 months. Hence, the follow-up data from patients attending the eHealth program reduced to n=49 at 3 months.

Participants were predominantly female (190/235, 81%) with a mean age of 38.3 (SD 12.4) years (Characteristics are summarized in Table 1). An unpaired *t* test showed that there was no difference in age between groups. A Mann-Whitney *U* test was performed to evaluate whether sex differed between groups, reveling no significant difference between patients attending the eHealth program and patients attending the IBS-school (Table S1 in Multimedia Appendix 1, *U*=6407.0, *z*=−1.394, *P*=.16). Furthermore, participants at the IBS-school and eHealth program displayed similar baseline characteristics upon enrollment, including moderate to severe IBS symptom severity.

There was no significant difference between most relevant features at baseline, except for anxiety and depression, as shown in Table 1. Here, patients attending the eHealth program scored an average of 11 (SD 5.2) which corresponds to mild to moderate anxiety, while patients attending the IBS-school scored 9.4 (SD 5.1), corresponding to a mild level of anxiety (2-tailed *t*_232_*=*2.37, *P*=.02). Thus, both patient groups displayed an anxiety score typical of a clinical case of anxiety (HADS-anxiety ≥8 is considered a clinical case [20]), and patients attending the eHealth program presented a significantly higher baseline score than patients attending the IBS-school. Furthermore, patients attending the eHealth program reported significantly higher depression scores than patients attending the IBS-school (mean 7, SD 4.1 vs mean 5.9, SD 4, respectively; *t*_232_=2.01, *P*=.045). However, both groups displayed an average depression score corresponding to nonclinical severity of depression (HADS-depression ≤8 is considered noncase [20]) which gives the difference little relevance on group level.

**Table 1.** Baseline patient characteristics and questionnaire data of 242 patients with irritable bowel syndrome (IBS) at enrollment to the eHealth program or IBS-school interventions. Unpaired *t* test unless otherwise stated; the reduced number from total number of participants indicate missing data.

		eHealth program		IBS-school		Significance	
		n	Mean (SD)	n	Mean (SD)	*t* test (*df*)	*P* value
Participant age		125	38.3 (12.4)	117	38.4 (13.4)	−0.06 (234)	.95
**Gender**							
	Men	27	—^a^	18	—	—	—
	Women	92	—	98	—	—	.16^b^
IBS symptom severity (IBS-SSS^c^)		125	282.7 (82.5)	117	268.5 (87.9)	1.29 (240)	.20
Anxiety (HADS-A^d^)		124	11 (5.2)	112	9.4 (5.1)	2.37 (232)	.02^e^
Depression (HADS-D^f^)		124	7 (4.1)	112	5.9 (4)	2.01 (232)	. 045 ^e^
**IBS-QOL** ^g^		123		117			
	Overall score		50.4 (21.9)		51.8 (21.0)	−0.48 (238)	.63
	Body image		41.0 (21.3)		44.6 (22.9)	−1.27 (238)	.21
	Dysphoria		47.0 (23.5)		50.0 (24.0)	−0.97 (238)	.33
	Food avoidance		29.0 (22.2)		28.3 (21.9)	0.25 (238)	.80
	Health worry		52.5 (22.9)		57.0 (23.0)	−1.46 (238)	.15
	Interference with activity		45.0 (21.9)		44.2 (21.3)	0.32 (238)	.90
	Relationships		56.7 (23.2)		57.6 (22.6)	−0.27 (238)	.79
	Social relations		51.6 (22.1)		54.3 (22.6)	−0.93 (238)	.35
	Sexual activity		57.8 (29.6)		57.8 (32.9)	−0.05 (238)	.96

a Not applicable.

b Mann-Whitney *U* test (Mean rank, *U*, *z* in Table S1 in Multimedia Appendix 1).

c IBS-SSS: Irritable Bowel Syndrome Symptom Severity Scale.

d HADS-A: Hospital Anxiety and Depression Scale – Anxiety.

e Significant at the *P*<.05 level.

f HADS-D: Hospital Anxiety and Depression Scale – Depression.

g IBS-QOL: irritable bowel syndrome quality of life.

### Symptom Relief and Enhanced Quality of Life

### Overview

**Figure 3.**
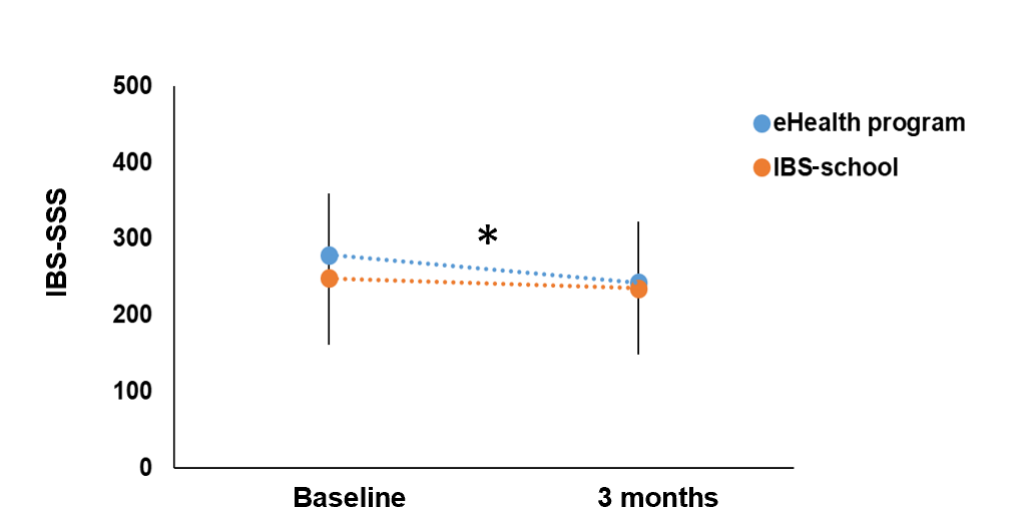
Overall change in IBS-SSS over time. On average, patients who completed the 3-month questionnaires at the IBS-school reported a 13 point reduction in IBS-SSS (n= 50), whereas eHealth program participants reported a 37-point reduction (n=71). A decrease of 50 points is defined as a clinical response to the intervention, categorizing the patient as a “responder to the intervention.” The IBS-SSS eHealth program score at baseline was 279 (SD 80) and 243 (SD 79) at 3 months, *P*<.001; the IBS-SSS IBS-school score at baseline was 248 (SD 87) and 235 (SD 99) at 3 months (*P*=.40). Paired *t* test results are summarized in Table S2 in Multimedia Appendix 1. *P<.001, indicating a significant difference in IBS-SSS scores before and after eHealth program intervention. Blue dot represents the eHealth program; orange dot represents the IBS-school. Paired *t* test was performed. Error bars present SD. Lower n indicate missing data. The reduced number from the total number of participants indicate missing data. IBS: irritable bowel syndrome. IBS-SSS: Irritable Bowel Syndrome Symptom Severity Scale.

A paired *t* test revealed that patients attending the eHealth program reported a significant reduction in IBS symptom severity at 3 months, shown in Figure 3 (mean 279.3, SD 80 vs mean 242.7, SD 79.3; *t*_70_=4.91, *P*<.001). Comparably, patients attending the IBS-school did not report a significant reduction in symptom severity (mean 248.6, SD 86.6 vs mean 235.4, SD 98.9; *t*_49_=0.85, *P*=.40). Refer to Table S2 of Multimedia Appendix 1 for data. However, none of the groups achieved a clinically meaningful improvement of ≥50 points on IBS-SSS at 3 months. Patients in the eHealth program-group reported a 37-point reduction, whereas patients attending IBS-school reported a 13-point reduction in IBS-SSS total score (Table S2 of Multimedia Appendix 1). Thus, for further analysis we categorized participants as a “responder” or “non-responder” to the intervention, where the sample response threshold for responders was set as ≥50-point decrease in total IBS-SSS score, a threshold that has been demonstrated to correlate with improvements in clinical symptoms [2].

#### Responders

Of the 71 patients, 29 (41%) attending the eHealth program responded to the intervention with a reduction in IBS-SSS score of ≥50 points, compared with 36% (18/50) who attended the IBS-school after 3 months (Figure 3). The results in this section are summarized in Tables2  3. Patients categorized as responders to the eHealth program reported a significant mean reduction in IBS-SSS score of 103 (SD 72.0) points with a moderate effect size of 0.56 (*t*_28_=7.62, Cohen *d*=1.33, *P*<.001). In addition, eHealth program–responding patients reported a significant reduction in anxiety, changing from a clinical case to a noncase classification (mean −4.7, SD 6.2; *t*_28_=6.89, Cohen *d*=0.85, *P*<.001). Here, the effect size of 0.39 indicated a moderate effect. On average, these patients also reported an increase in overall quality of life, summarized in Tables2  3 (mean 5.80, SD 19.1; *t*_28_*=*−2.28, Cohen *d*=−0.30, *P*=.03). Here, features such as dysphoria (mean 18, SD 23.3; *t*_28_*=*−2.1, Cohen *d*=−0.85, *P*<.001) and body image (mean 14.8, SD 18.6; *t*_28_=−3.9, Cohen *d*=−0.72, *P*<.001) increased significantly with a moderate effect size. Features such as food avoidance (mean 6.3, SD 22.5; *t*_28_=−2.10, Cohen *d*=−0.13, *P=*.045), health worry (mean 10.8, SD 23.5; *t*_28_=−3.44, Cohen *d*=−0.43, *P*=.002), interference with activity (mean 13.4, SD 18.8; *t*_28_=−4.03, Cohen *d*=−0.63, *P*<.001), relations (mean 9.5, SD 22.9; *t*_28_=−2.72, Cohen *d*=−0.37, *P*=.01), and social relations (mean 10.7, SD 19.4; *t*_28_=−3.62, Cohen *d*=−0.56, *P*=.001), all improved significantly, compared with baseline. The changes in sexual activity were not significantly different after 3 months (mean 5.4, SD 29.8; *t*_28_=−1.52, Cohen *d*=−0.19, *P*=.14).

**Table 2.** Changes in symptom severity, anxiety and depression, and quality of life 3 months after the eHealth program in patients with irritable bowel syndrome (IBS) who attended the eHealth program. Lower n indicate missing data; the reduced number from the total number of participants indicate missing data.

		Responder						Nonresponder					
		n	mean (SD)	*t* test (*df*)	Cohen *d*	Effect size *r*	*P* value	n	mean (SD)	*t* test (*df*)	Cohen *d*	Effect size *r*	*P* value
IBS symptom severity(IBS-SSS^a^)		29	−102.8 (72.0)	7.62 (28)	1.33	0.56	<.001^b^	42	9.2 (70.4)	−1.73 (41)	−0.13	−0.06	.09
Anxiety (HADS-A^c^)		29	−4.7 (6.2)	6.80 (28)	0.85	0.39	<.001^b^	39	−2.4 (4.8)	3.65 (38)	0.52	0.25	.001^b^
Depression (HADS-D^d^)		29	−3.0 (4.6)	3.47 (28)	0.76	0.36	<.001^b^	39	−1.3 (3.8)	1.89 (38)	0.34	0.17	.67
**IBS-QOL** ^e^ **, overall score**		29	5.80 (19.1)	−2.28 (28)	−0.30	−0.15	.03^b^	37	2.5 (24.8)	−0.79 (36)	−0.10	−0.05	.44
	Body image		14.8 (18.6)	−3.90 (28)	−0.72	−0.34	.001^b^		3.8 (25.4)	−1.24 (36)	−0.16	−0.08	.15
	Dysphoria		18.0 (23.3)	−2.10 (28)	−0.85	−0.39	<.001^b^		2.8 (24.3)	−1.20 (36)	−0.11	−0.05	.24
	Food avoidance		6.3 (22.5)	−2.10 (28)	−0.26	−0.13	.045^b^		4.8 (21.7)	−1.94 (36)	−0.22	−0.11	.06
	Health worry		10.8 (23.5)	−3.44 (28)	−0.43	−0.21	.002^b^		1.8 (23.5)	−0.60 (36)	−0.08	−0.04	.55
	Interference with activity		13.4 (18.8)	−4.03 (28)	−0.63	−0.30	<.001^b^		2.5 (23.65)	−1.11 (36)	−0.11	−0.05	.28
	Relationships		9.5 (22.9)	−2.72 (28)	−0.37	−0.18	.01^b^		4.6 (21)	−1.81 (36)	−0.20	−0.10	.08
	Social relations		10.7 (19.4)	−3.62 (28)	−0.56	−0.27	.001^b^		10.7 (24.6)	−0.53 (36)	−0.46	−0.23	.60
	Sexual activity		5.4 (29.8)	−1.52 (28)	−0.19	−0.10	0.14		5.4 (30.13)	−0.56 (36)	−0.19	−0.09	.58

a IBS-SSS: Irritable Bowel Syndrome Symptom Severity Scale.

b Significant at the *P*<.05 level.

c HADS-A: Hospital Anxiety and Depression Scale – Anxiety.

d HADS-D: Hospital Anxiety and Depression Scale – Depression.

e IBS-QOL: irritable bowel syndrome quality of life.

**Table 3.** Changes in symptom severity, anxiety and depression, and quality of life in patients with irritable bowel syndrome (IBS) 3 months after the IBS-school. Lower n indicate missing data; the reduced number from the total number of participants indicate missing data.

		Responder						Nonresponder					
		n	mean (SD)	*t* test (*df*)	Cohen *d*	Effect size *r*	*P* value	n	mean (SD)	*t* test (*df*)	Cohen *d*	Effect size *r*	*P* value
IBS symptom severity(IBS-SSS)^a^		18	−119.3 (86.2)	5.45 (17)	1.33	0.56	<.001^b^	32	46.5 (77.8)	−4.08 (31)	−0.57	−0.27	<.001^b^
Anxiety (HADS-A^c^)		16	−2.5 (6.8)	1.97 (15)	0.43	0.21	.07	29	−1.9 (5.3)	2.02 (28)	0.38	0.19	.05
Depression (HADS-D^d^)		16	−2.3 (4.4)	2.17 (15)	0.55	0.27	.046^b^	29	−0.5 (3.9)	0.56 (28)	0.12	0.06	.58
**IBS-QOL** ^e^ **, overall score**		8	2.4 (27.9)	−0.49 (7)	−0.08	−0.04	.64	24	−11.4 (23.8)	3.16 (23)	5.52	0.25	.004^b^
	Body image		1.68 (26.5)	−0.57 (7)	−0.05	−0.02	.59		−4.0 (22.4)	1.38 (23)	0.18	0.09	.18
	Dysphoria		17 (27.2)	−6.76 (7)	−0.62	−0.30	<.001^b^		−3.3 (21.7)	1.11 (23)	0.15	0.08	.28
	Food avoidance		5.3 (28)	−0.62 (7)	−0.17	−0.09	.56		0.8 (21.6)	−0.13 (23)	−0.03	−0.02	.85
	Health worry		13.7 (15.3)	−2.76 (7)	−0.67	−0.32	.03^b^		0 (22.4)	−0.01 (23)	0	0	.20
	Interference with activity		9.3 (28.1)	−3.14 (7)	−0.30	−0.15	.02^b^		−0.5 (19.1)	0.19 (23)	0.02	0.01	.85
	Relationships		21.2 (22.6)	−3.50 (7)	−0.87	−0.40	.01^b^		18.7 (17)	−2.11 (23)	−0.86	−0.39	.046^b^
	Social relations		7.8 (21.4)	−1.23 (7)	−0.27	−0.13	.26		6.4 (24.2)	1.84 (23)	0.26	0.13	.08
	Sexual activity		5.4 (29.8)	1.05 (7)	−0.19	−0.10	.33		2.8 (31.6)	0.81 (23)	0.08	0.04	.43

a IBS-SSS: Irritable Bowel Syndrome Symptom Severity Scale.

b Significant at the *P*<.05 level.

c HADS-A: Hospital Anxiety and Depression Scale – Anxiety.

d HADS-D: Hospital Anxiety and Depression Scale – Depression.

e IBS-QOL: irritable bowel syndrome quality of life.

Patients who responded to the IBS-school intervention reported an average reduction in IBS symptom severity score of 119 (SD 86.2) points (n=18; *t*_17_=5.54), Cohen *d*=1.33, *P*<.001). In addition, patients reported a reduction in depression scores (mean −2.3, SD 4.4; *t*_15_=2.17, Cohen *d*, *P*=.046). However, these patients did not report significant improvements in anxiety or overall quality of life (mean 2.5, SD 6.8; *t*_15_=1.97, Cohen *d*=0.43, *P*=.07 and mean 2.4, SD 27.9; *t*_7_=−0.57, Cohen *d*=−0.08, *P*=.64, respectively). Furthermore, the number of responding patients were too low for further in-depth statistical analysis of quality of life (n=8). Data for these analyses are presented in Table S3 of Multimedia Appendix 1.

#### Nonresponders

Patients who were nonresponders to the eHealth program reported no improvement in symptom severity or quality of life (mean 9.2, SD 70.4; *t*_41_=−1.73, Cohen *d*=−0.13, *P*=.09) and a significant decrease in anxiety scores (mean −2.4, SD 4.8, *t*_38_=3.65, Cohen *d*=0.52, *P*=.001), compared with baseline. However, both changes were of small effect (*r*=−0.27 and 0.25, respectively). Results are summarized in Tables2  3. There were no significant changes in depression scores or overall quality of life or the respective sub-categories (*P*>.05). Nonresponding patients attending IBS-school reported significantly enhanced symptom scores (mean 46.5, SD 77.8; *t*_31_=−4.08, Cohen *d*=−0.57, *P*<.001). However, it is worth noting that these enhanced symptoms scores were not above the 50-point threshold for clinical relevance. Furthermore, these patients reported a significantly reduced overall quality of life (mean −11.4, SD 23.8; *t*_23_=3.16, Cohen *d*=5.52, *P*=.004) and a significant increase in the domain of relationships (mean 18.7, SD 17; *t*_23_=−2.11, Cohen *d*=−0.86, *P*=.046). Baseline characteristics for these analyses are presented in Table S3 of Multimedia Appendix 1.

#### Association Between Type of Intervention and Outcome

A *χ*^*2*^ test of independence analysis was used to test whether the type of intervention was independent from the intervention outcome. Results showed that the proportion of patients who responded to the intervention in both groups (29/71 vs 18/50) were the same. Hence, there was no significant association between the type of intervention and response to the intervention (Pearson *χ*^2^_1_=0.2, Φ=0.041, *P*=.65). Thus, we conclude that there is no difference in treatment response between the eHealth program and IBS-school in affecting symptom severity scores. Data from this analysis is reported in Table S4 of Multimedia Appendix 1.

### Patient Satisfaction

Patient satisfaction was investigated in both groups 3 months after enrollment (CSQ-8). Patients who attended the IBS-school reported a mean score of 24.2 (SD 3.7), compared with patients attending the eHealth program with a mean score of 23.5 (SD 4.0), which are both equivalent to “good” health care offers [22]. An unpaired *t* test revealed no significant difference between group scores (*t*_115_*=*−1.032, *P*=.31; Table 4).

**Table 4.** Difference in client satisfaction scores (CSQ-8) between patients attending the eHealth program and irritable bowel syndrome (IBS)–school. A *χ*^*2*^ test of independence was conducted. The reduced number from the total number of participants indicate missing data.

		n	mean (SD)	*t* test (*df*)	Cohen *d*	Effect size r	*P* value
**Overall**				−1.032 (115)	−0.193	−0.01	.31
	eHealth program	68	23.46 (4.0)				
	IBS-school	49	24.20 (3.67)				

## Discussion

### Principal Findings

In this study, we have shown that the novel digital multidisciplinary eHealth program has a significant reducing effect on IBS symptom severity and is useful as a tool in disease self-management. In total, 41% (29/71) of participants reported significant and clinically relevant symptom relief. Furthermore, we show that the eHealth program is safe, as patients not responding to the intervention reported unchanged symptoms and quality of life at 3 months. In addition, eHealth intervention–responding patients reported significant benefits on multiple domains of IBS-related quality of life such as body image, food avoidance, health worry, interference with activity, relations, and social relations. Levels of anxiety were significantly reduced, and levels of dysphoria were improved.

Comparably, 36% (18/50) of participants reported a clinically significant effect to the onsite multidisciplinary 2-day group-based education program, the so-called “IBS-school.” Thus, our results indicate that the digital multidisciplinary eHealth program may be equally effective to the IBS-school, which is often a standard treatment offer to newly diagnosed patients. Furthermore, patients responding to the IBS-school intervention did not report any significant improvements in quality of life or in anxiety, but a small not clinically meaningful decrease in depression scores. The number of IBS-school responders were too low for more in-depth statistical analysis on the domains of quality of life.

A *χ*^*2*^ test of independence showed no difference between intervention outcome in the 2 groups; hence the eHealth program did not have a better intervention response than the IBS-school on the measures of symptom severity. However, the eHealth program had a significant effect on other aspects of IBS symptomatology, including anxiety and quality of life, whereas IBS-school did not.

Patients rated both the eHealth program and the IBS-school as good health care offers on measures of patient satisfaction of health care quality, scoring them 23.5 and 24.2 out of a maximum of 32 points, respectively.

We believe these results indicate that a digital eHealth approach, designed to provide patients with evidence-based information and practical skills, is preferable to an onsite multidisciplinary 2-day group-based education program covering the same topics.

### Comparison With Previous Work

Initially at baseline in both groups, the greatest impairment in quality of life was observed for the subscale of food avoidance followed by body image, inactivity, and dysphoria. This order of impairment is similar to findings by Drossmann et al [21] in an international study from 2009 (n=1966). However, our findings show lower scores on all subscales except dysphoria and health worries, which are in the same magnitude. In comparison with a newer study on quality of life by Kopczyńska et al [23] (n=87), our baseline results show much lower scores on both overall and all subscales of quality of life. A recent Vietnamese study showed that patient education, lifestyle, and dietary intervention, administered by clinical pharmacists, improved IBS related quality of life compared with standard medical therapy over 8 weeks [10]. These study patients also showed a much higher baseline and postintervention quality of life-scores compared with our study. We speculate that our study participants represent a group with more severe IBS because they are referred to a tertiary health care institution that due to capacity issues has to prioritize those patients who need it the most. However, the low baseline scores on food avoidance in our study indicate that both our interventions with a key focus on diet was the right call. This core problem was targeted because food is a known important trigger of IBS-symptoms [24]. On the domain of food avoidance, we observed an improvement in eHealth program-responders, compared with baseline (Tables2  3). No such improvement was observed in patients attending the IBS-school. We speculate that this is due to the differences in comprehensiveness and durability. The eHealth program is designed to engage the patient interactively to learn how to make appropriate changes in diet. It offers a thorough guidance with videos and instructions on how to follow the low FODMAP diet, including the exclusion and reintroduction of foods. In addition, the patient has the option of digital question and answer sessions with a clinical dietitian throughout the program. The IBS-school is also designed for patients to learn how to self-help, but the program is much shorter with its 2 days of physical attendance. In the light of these results, we deduce that the eHealth program is not inferior to the established IBS-school as a health care offer. In fact, we show that an eHealth intervention program can provide the patient with self-help tools that can lead to reduced gastrointestinal symptoms and enhance quality of life for patients with IBS. This aligns with a recent randomized controlled study by Tayama et al [13] (n=40) showing that a multidisciplinary eHealth self-management program leads to an increased intake of FODMAP-groups and subsequently a more extensive diet. Severe food avoidance and dietary restriction is previously reported in 13% (829/955) of IBS-patients and this subgroup of IBS-patients reported more severe IBS-symptoms, reduced quality of life, and reduced intake of nutrients [24]. Thus, providing patients with evidence-based information, practical tools, and support by a clinical dietitian is important in clinical care of patients with IBS.

eHealth programs in the form of apps, internet-guided programs, or telehealth has recently accelerated as useful tools in clinical medicine. In an American study on satisfaction during COVID-19, most patients with IBS reported high satisfaction rates and ease of use with telehealth [25]. Here the authors reported on multiple benefits including the patient having to take less time off from work and improved access to the care team. Their most commonly reported challenges with telehealth included feeling impersonal and being unable to address all of their issues or concerns. A majority felt that telehealth was as good as or better than face-to-face visits and would use telehealth for future care. Only approximately 10% (130/1311) of the patients remained dissatisfied. In 2020, a Polish study showed that an educational program combined with elements of behavioral therapy, individualized for patients with IBS, is an important part of therapy [26]. In addition, as a part of a dietetic-led gastroenterology service in primary care, feasibility, acceptability, and cost-efficiency of using webinars to deliver first-line patient education for patients with IBS, has been shown to be successful [27]. A meta-analysis of chronic gastrointestinal illness interventions (19 studies conducted in 8 countries, n=3193) showed that eHealth gastrointestinal interventions improved patients’ quality of life, psychological distress, medication adherence, and illness-related knowledge [28]. The meta-analysis also showed that eHealth gastrointestinal interventions significantly reduced the number of patient visits to the hospital. Taken together with our results, these findings support eHealth interventions holding decent promise in improving outcomes for patients with IBS.

Comparably on patient satisfaction, a randomized controlled trial by Lackner et al [29] showed that patients who received 4 gastroenterologist-led patient education sessions over 2 weeks reported 26.6 in patient satisfaction. Here, 43.5% (63/145) of patients reported improvement in addition to a higher satisfaction score than in our study. However, both these and our results reflect that subjective symptom relief may not be required for the patient to experience the treatment as useful. This is also highlighted by our patients attending the IBS-school who did not experience significant enhancement in quality of life nor reduction in symptom severity, but still reported a higher satisfaction than patients who objectively benefited more from the eHealth program.

### Limitations

All 255 recruited patients completed the programs. However, there was a very high percentage that did not submit the 3-month questionnaires. In total, 41% (54/132) percent of patients attending the eHealth program, and 54% (67/123) of the patients who attended the IBS-school did not submit the 3-month follow-up questionnaires on IBS symptoms severity (Figure 1). Unfortunately, this occurred despite multiple efforts and reminders (phone calls, emails, and SMS text messages) from the research team encouraging the participants to respond. First, this high percentage of missing data are significant and may have affected the results of the study even though the rates appear similar across the groups. However, high dropout rates are a common issue in eHealth studies and known as “the law of attrition” [30]. This warrants the need for a larger study where an intention-to-treat analysis can be carried out without diminishing the power of the study. Second, eHealth literacy is defined by Norman and Skinner [31] as “the ability to seek, find, understand, and appraise health information from electronic sources and apply the knowledge gained to addressing or solving a health problem”. The individual patient’s level of health literacy was not mapped during the study and variations in these abilities may have affected our results. Third, the patient population represents all subtypes of IBS and there are no analyses focusing on the differences in response between patients with predominant diarrhea, constipation, or a mix of the 2. Comorbidities or other additional diagnoses are common in IBS [32] but have not been excluded in this study and may have affected the results. Fourth, the use of drugs during the study period have not been reported. Hence, many drugs have side effects such as nausea, vomiting, diarrhea, constipation, flatulence, which are symptoms that the patient may confused with IBS symptoms. Fifth, a high placebo effect, which can be up to 40%, is a known challenge in clinical studies on IBS [20]. Although this study has not been designed with a control group, the placebo effect may have affected our results in either group and have not been adjusted for. However, we may speculate that the phenomenon has affected patients in both groups equally and importantly, the placebo effect may recede after 12 weeks, which was our end point [33]. Sixth, as both programs are broad and cover a variety of information, advice, and treatment including the low FODMAP diet and principles of CBT, another limitation of this study is the unknown specifics patients were responding to in either program. Indeed, there are no measures on cognition verifying the direct effects of the principles of CBT or exposure therapy. This needs to be further investigated in a prospective study, and we acknowledge that an in-depth explanation for our observed benefits after attending the eHealth program remain to be clarified. Thus, these aspects are objectives in our currently ongoing randomized controlled trial [34]. In the light of limited primary and secondary health care resources, it will be useful to develop prediction tools to identify which patients may achieve improvement in both symptom severity and domains of quality of life. For these stratification analyses to be clinically meaningful, the number of participants need to be higher than reported in this study and performed in and randomized controlled trial.

### Conclusions

We conclude that the digital multidisciplinary eHealth program has a significant effect on IBS symptom severity in a portion of patients, and is useful as a tool in disease self-management. In addition, it does not result in worse symptom scores than an onsite multidisciplinary 2-day group-based education program after 3 months. We believe these results indicate that a digital eHealth approach, that include benefits such as 3 months unlimited access to quality assured information and treatment with documented effect, is preferable to an onsite multidisciplinary 2-day group-based education program covering the same topics.

## Supplementary material

10.2196/43618 Multimedia Appendix 1 Supplementary tables.

## Abbreviations

CBT: cognitive behavioral therapy
CSQ-8: client satisfaction questionnaire
FODMAP: fermentable oligosaccharides, disaccharides, monosaccharides, and polyols
HADS: Hospital Anxiety and Depression Score
IBS: irritable bowel syndrome
IBS-QOL: irritable bowel syndrome quality of life
IBS-SSS: Irritable Bowel Syndrome Symptom Severity Scale
*ICD-10*: *International Statistical Classification of Diseases, Tenth Revision*
*ICPC-2*: *International Classification of Primary Care, Second Edition*
